# Implementation of radiation therapist cylinder insertion for vaginal vault brachytherapy

**DOI:** 10.1002/jmrs.329

**Published:** 2019-03-04

**Authors:** Dean B. Paterson, Shelley M. Pearson, Carol A. Johnson

**Affiliations:** ^1^ Radiation Treatment Department, Blood and Cancer Centre Wellington Regional Hospital Wellington New Zealand

## Abstract

Vaginal vault brachytherapy is a common treatment for endometrial cancer. Historically, applicator insertion has been the domain of a radiation oncologist (RO). This commentary outlines a project to improve efficiency and workforce utilisation by introducing a competency framework and training module allowing entitled radiation therapists to perform single‐channel cylinder applicator insertions and treatment delivery under RO supervision for fraction one and without supervision for subsequent fractions. The rationale, relevant regulations, implementation process and barriers are explored.

## Introduction

**Figure 1:**
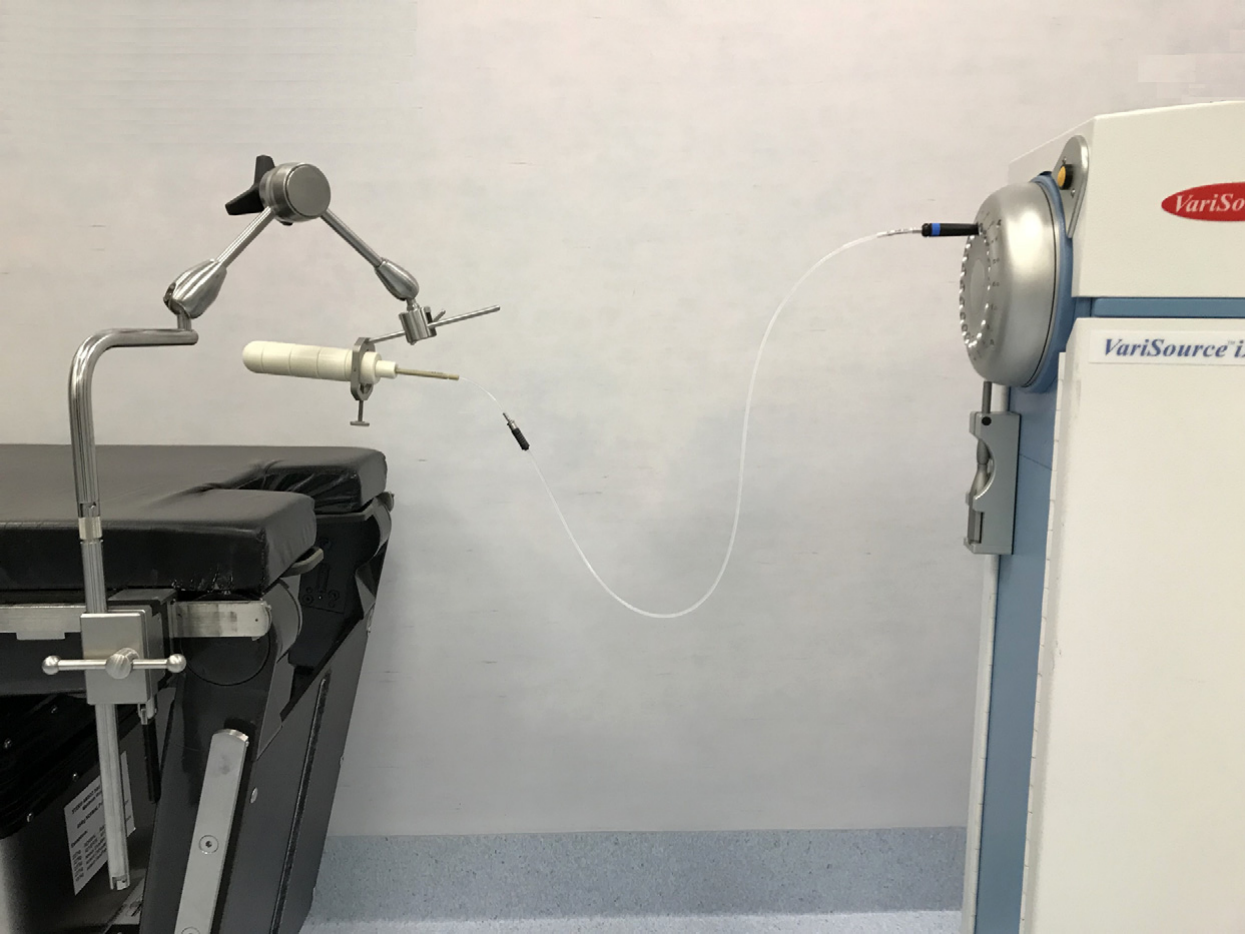
Cylinder applicator connected to a high‐dose‐rate (HDR) brachytherapy remote afterloader containing a radioactive Iridium‐192 source wire. [Colour figure can be viewed at wileyonlinelibrary.com]

Endometrial cancer is the most prevalent gynaecological cancer in New Zealand (NZ).^1^ High‐dose‐rate (HDR) vaginal vault brachytherapy (VVBT) is a common treatment for endometrial cancer that aims to reduce the risk of local recurrence post‐hysterectomy. It can be delivered alone or in combination with external beam radiation therapy depending on the stage and grade of disease.^2^ A single‐channel cylinder is the most common VVBT applicator.^3^ Typical regimens involve two to four fractions, during which the applicator is placed intra‐vaginally. An Iridium‐192 radioactive source is then positioned inside the applicator at pre‐specified locations and times using a remote afterloader (Fig. 1).

Historically, the role of radiation therapists (RTs) for VVBT at our institution was in treatment planning and afterloader operation. The radiation oncologist (RO) performed the insertion or supervised their radiation oncology registrar to perform this task. In addition, the RO was required to be present at each treatment and was designated first responder in the event of a radiation emergency.

This commentary outlines the implementation of RT‐led applicator insertion and treatment delivery for VVBT at the Wellington Blood and Cancer Centre. The aim of this project was to improve brachytherapy unit efficiency and workforce utilisation by introducing a competency framework and training module to allow entitled RTs to perform cylinder insertions and treatment delivery under RO supervision for fraction one and without supervision for subsequent fractions.

## Rationale

Trained RTs operating within a defined competency framework are well‐placed to perform some tasks that have previously been the domain of physicians.^4^, ^5^, ^6^ RT cylinder insertion and treatment delivery for VVBT has been implemented at a number of National Health Service (NHS) hospitals in the United Kingdom (UK).^4^, ^5^, ^7^, ^8^, ^9^, ^10^, ^11^ The Royal College of Radiologists^6^ states that “vault brachytherapy applicators may be inserted by appropriately trained clinicians, nurses or radiographers after assessment by a specialised clinical oncologist and under their supervision” (pg. 7). In some UK centres, radiographers have also been trained to perform vaginal exams,^8^ applicator sizing^8^, ^9^ and consent.^8^ The introduction of an RT‐led service has been reported to improve efficiency,^8^, ^9^, ^11^ patient care,^8^, ^11^ workforce utilisation^9^, ^11^ and job satisfaction for RTs.^9^, ^11^

The following limitations existed at our institution due to the requirement of treatment appointments to be booked around RO availability:

1 Inefficient use of brachytherapy unit
  a. treatment limited to 2 days per week
  b. scheduling conflicts with cervical brachytherapy which occurs on the same days as VVBT (57.5% of cases)
  c. patient throughput limited to three per week due to applicator availability
  d. inability to offer appointment flexibility
2 Inefficient workforce utilisation
  a. heavy workload for ROs who could be better utilised for more complex tasks
  b. RO often double‐booked and patients must wait for RO to be available
  c. additional RTs are rostered to the brachytherapy unit when VVBT and cervix brachytherapy are scheduled on the same day

A 48% increase in VVBT treatments between 2014 (54 fractions) and 2017 (80 fractions) at our institution expedited the need to improve workflow efficiency. Prior to this, RT cylinder insertion has not been performed at any of the five departments that perform VVBT in NZ (National Brachytherapy Workshop, 23 September 2017).

## Regulation

The NZ Medical Radiation Technologist Board (MRTB) were consulted to ensure that the proposed process aligned with the RT Scope of Practice and Competency Standards. According to the Competencies, RTs are responsible for the implementation and delivery of radiation treatment according to the approved plan/prescription of the RO as well as safely managing radiation/radioactivity in the workplace, including for brachytherapy.^12^

There is no requirement in the NZ Office of Radiation Safety's *Code of Safe Practice for the use of sealed radioactive materials for brachytherapy (C14)* that a RO must be present for HDR brachytherapy treatment.^13^ In addition, a precedent already existed at our institution for surface mould brachytherapy where the applicator is placed by RTs. The RO is not required to verify mould placement or be present for treatment as these applicators are deemed “easy to remove” in the event of a radiation emergency. Cylinders are also “easy to remove” without specialist knowledge. In summary a RT with specialist education and training, according to a defined competency framework, can perform radiological procedures on delegation from the consultant.

The Royal Australasian and NZ College of Radiologists (RANZCR) gynaecological special interest group was consulted prior to implementation. Feedback regarding the number of supervised insertions required was incorporated into the training module. The proposal was also approved by the Capital and Coast District Health Board Clinical Practice Committee.

## Implementation

A project group was set up consisting of the Head of Treatment, initial RT trainee and the Clinical Leader RO. The project scope was determined and is outlined in Table 1.

**Table 1:** RT‐led cylinder insertion project scope

In scope	Out of scope
Single‐channel cylinder insertion for VVBT Under RO supervision for fraction one Without direct supervision for subsequent treatment fractions Removal of requirement for RO to be present for treatment delivery	Applicator sizing procedure Multi‐channel cylinder insertion Ovoid applicator insertion Patients deemed unsuitable for RT‐led cylinder insertion

**Figure 2:**
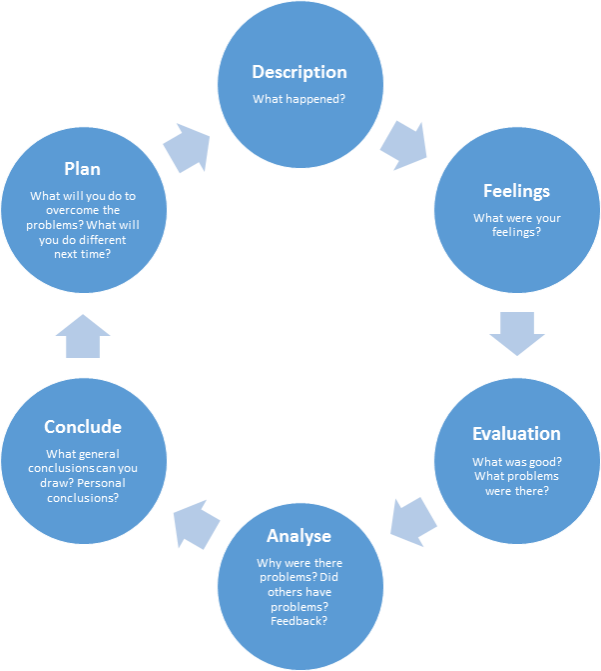
Gibbs’ reflective cycle.^14^

The group developed a competency framework, adapted from that used at Guy's and St Thomas’ NHS Foundation Trust (Caroline Nahab – Advanced Practitioner Radiographer, email correspondence, 2017 January 5), on which to base training (Table 2). A training module was created to address each of the competencies. This included required readings as well as the successful completion of 20 cylinder insertions under direct RO guidance and supervision. Initially the framework only required 15 supervised insertions however this increased to 20 following feedback on the initial proposal by the RANZCR gynaecological special interest group. A reflective case‐study was required for each insertion, which the trainee used to demonstrate an understanding of the literature included in the module as well as reflect on their practice. Reflective practice enables the practitioner to plan for what they might do differently in the future in order to improve practice and is one of the key competencies for RTs in NZ.^12^ A template for a reflective case‐study was created based on The Gibbs’ Reflective Cycle^14^ (Fig. 2). This model is used by healthcare professionals worldwide and the reflection prompts for the Society of Radiographers (UK) continued professional development are based on this.^15^

**Table 2:** Summary of competency framework and training package content for RT‐led cylinder applicator insertion

Competency^1^	Description	Summary of training content
1	Understand current clinical practice and management of patients with endometrial cancer locally and internationally and how these align	Compulsory readings as well as a summary of each. Contains a section outlining how the Institution's clinical protocol aligns with the literature. Compulsory readings:^17^, ^18^, ^19^, ^20^, ^21^, ^22^
2	Understand current expert body recommendations	Contains a summary of the American Brachytherapy Society (ABS) and Groupe Européen de Curiethérapie and the European Society for Radiotherapy & Oncology (GEC‐ESTRO) recommendations on VVBT. Compulsory readings:^3^, ^23^, ^24^
3	Understand regional referral pathway	Process at Institution is outlined.
4	Understand female pelvic anatomy before and after surgery	Describes surgical pathway based on disease stage and grade at our Institution. Contains anatomical diagrams and CT images of female pelvic anatomy pre and post‐surgery.
5	Understand possible side effects and ways in which these can be managed a. Demonstrate understanding of when to refer to RO b. Side effects related to brachytherapy and surgery	Covers reported rates of treatment related side‐effects. Describes the symptoms, causes, assessment, management and referral criteria for various side‐effects: Bladder Bowel (diarrhoea and constipation) Vaginal Infection Pain Prolapse Menopause
6	Understand the importance of correct size and placement of applicator	Covers information on the range of applicators available (locally and internationally), effect of air gaps,^23^, ^25^ reports on incorrect treatment delivery in cylinder based VVBT^26^ and describes the insertion process.^23^, ^24^, ^26^
7	Successful completion of 20 vaginal cylinder insertions under direct RO guidance and supervision	The RO will perform the first insertion and describe their technique to the RT. The RT will then perform the subsequent insertions for that patient. It is therefore anticipated that the RT will have experience performing cylinder insertions on a minimum of seven patients prior to sign‐off.
8	Complete a reflective case study for each training insertion	Reflective case study template created based on Gibbs’ Reflective Cycle^14^

^1^ Adapted from Guy's and St Thomas’ NHS Foundation Trust, London, United Kingdom.

On completion of training, the RT is considered competent to accept the delegated responsibilities of VVBT cylinder insertions. The final decision of delegation is made by the Clinical Leader.

Initially, only one RT has been trained and is entitled to perform cylinder insertions, however, it is expected that this could be rolled out to more RTs at a later stage. A pre‐requisite for training is completion of the post‐graduate paper ‘RADX403: Brachytherapy Principles and Practice’ from the University of Otago, Wellington (or equivalent). The RT must complete at least 15 insertions per year to maintain competency.

Training for the initial RT occurred between January and October 2018. During this time 27 patients received VVBT of which 12 (44%) was with a single‐channel cylinder. A reflective case‐study was completed for each insertion and reviewed by the supervising RO with the trainee. The process worked well with no issues identified by the trainee, the Clinical Leader or patients. The trainee reported that the training programme provided an excellent foundation to build clinical experience and is now entitled to perform cylinder insertions from fraction two onwards without direct supervision.

## Barriers

**Figure 3:**
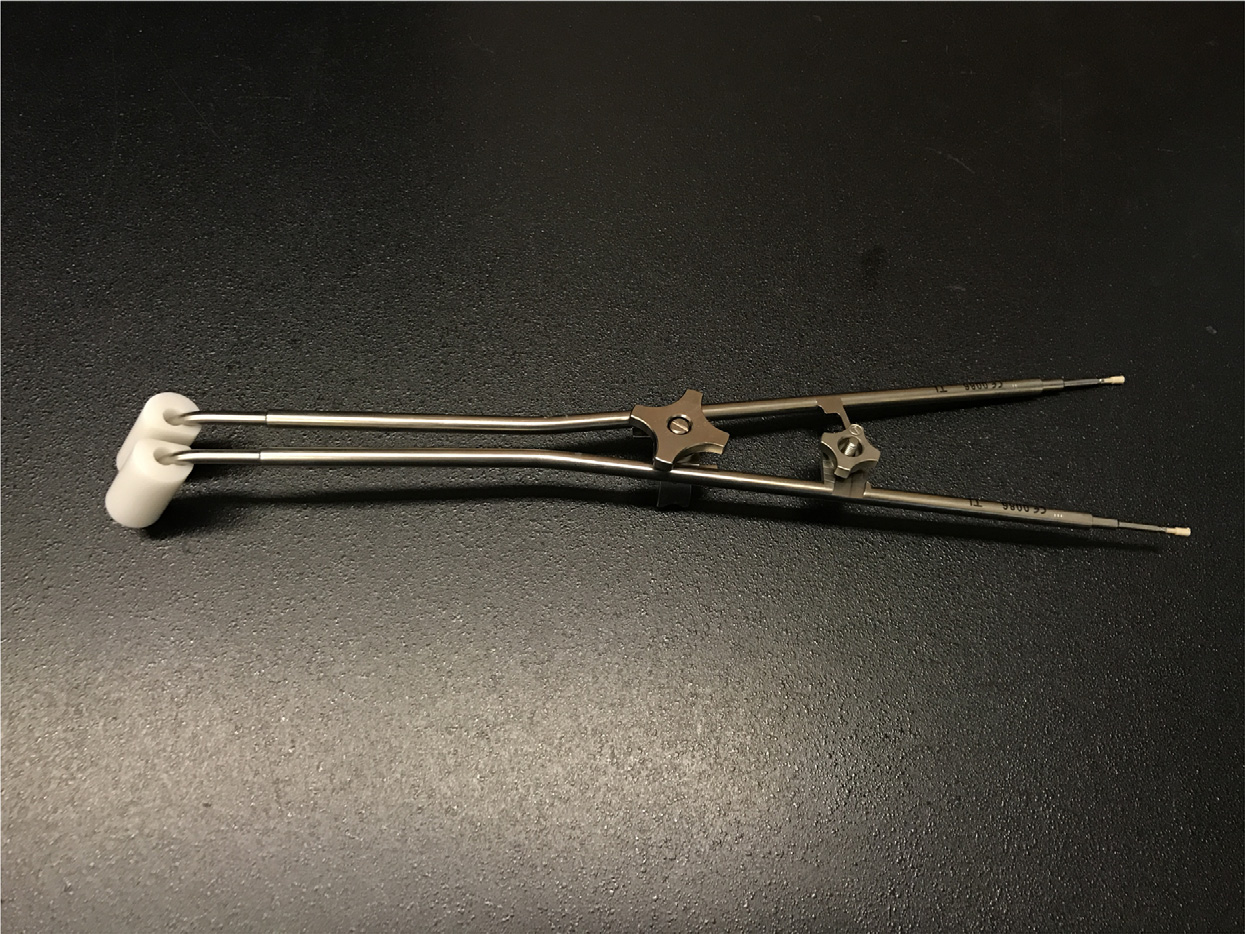
Ovoids applicator configured for vaginal vault brachytherapy.

The main barrier to the trainee RT gaining clinical experience with 20 supervised insertions was the proportion of patients requiring vaginal ovoids, rather than a cylinder applicator. In 2016, this proportion was 18% however this increased to 56% in 2018. Although the cylinder is the preferred applicator at our institution (due to simplicity of use, insertion and planning), ovoids (Fig. 3) are used to ameliorate the effect of “dog‐ears” when present on the planning CT. “Dog‐ears” are a post‐operative anatomical configuration resulting in an expansion in the lateral apices because of remnants of the vaginal fornices.^16^ With any VVBT technique, careful attention must be paid to maintaining conformity between the applicator surface and the vaginal mucosa, particularly at the vault. Although ovoids have better conformity in the presence of “dog‐ears”, there is no clinical evidence that recurrences are reduced due to their use.^16^

Another barrier was scheduling VVBT on busy brachytherapy days when RT staff are required for other tasks, such as planning cervical brachytherapy cases. This was anticipated as scheduling conflicts and efficiency gains were the primary factors driving this project. On a number of occasions the trainee could not perform insertions due to other responsibilities.

There was concern that radiation oncology registrar training opportunities would be limited if RTs perform the majority of VVBT insertions. At our institution brachytherapy is an essential part of radiation oncology registrar training. This concern was addressed by involving radiation oncology registrars in the applicator sizing procedure and empowering them to identify specific cases that can be reserved for their own training.

## Future Direction

Planning is underway to expand RT training to include the insertion of ovoids. This will significantly increase the proportion of cases that are RT‐led and will further support our institution in achieving improved efficiency and workforce utilisation. Ovoids are classified as an “easy to remove” applicator in the event of a radiation emergency and RTs are already well‐versed in the assembly and use of this applicator having written the local documentation on its use.

The existing training programme can be adapted for ovoids (i.e. the successful completion of 20 supervised ovoid insertions with reflection) and the content does not require significant changes – this already covers the range of applicators available and when each type is indicated.

## Conclusion

A competency framework and training module has been implemented to enable entitled RTs to perform VVBT cylinder insertions and treatment delivery. Entitled RTs insert single‐channel cylinder applicators under RO supervision for fraction one and without supervision for subsequent fractions. It is anticipated that this process will increase efficiency and workforce utilisation. Work is ongoing to expand this programme to include ovoid insertions and the continual monitoring of efficiency outcomes.

## Conflict of Interest

The authors declare no conflict of interest.
